# Synergistic Photocatalytic-Adsorption Removal of Basic Magenta Effect of AgZnO/Polyoxometalates Nanocomposites

**DOI:** 10.1186/s11671-021-03620-0

**Published:** 2021-11-10

**Authors:** Heyun Tian, Jie Luo, Ke Zhang, Chenguang Ma, Yiyi Qi, Shixia Zhan, Xiao Liu, Mingxue Li, Hongling Liu

**Affiliations:** Key Lab of Polyoxometalate Chemistry of Henan Province, Institute of Molecular and Crystal Engineering, School of Chemistry and Chemical Engineering, Henan University, Kaifeng, 475001 China

**Keywords:** AgZnO/polyoxometalates, Nanocomposites, Photocatalytic, Adsorption, Basic magenta removal

## Abstract

The bifunctional photocatalytic-adsorbent AgZnO/polyoxometalates (AgZnO/POMs) nanocomposites were synthesized by combining AgZnO hybrid nanoparticles and polyoxometalates [Cu(L)_2_(H_2_O)]H_2_[Cu(L)_2_(P_2_Mo_5_O_23_)]⋅4H_2_O (HL = C_6_H_6_N_2_O) into nanostructures via a sonochemical method. Transmission electron microscopy (TEM) indicated that AgZnO/POMs nanocomposites were uniform with narrow particle size distribution and without agglomeration. X-ray powder diffraction (XRD) and X-ray photoelectron spectroscopy (XPS) analysis confirmed the nanostructure and composition of AgZnO/POMs nanocomposites. The ultraviolet–visible spectra (UV–Vis) and photoluminescence spectra (PL) confirmed excellent optical properties of the AgZnO/POMs nanocomposites. 94.13% ± 0.61 of basic magenta (BM) in aqueous solution could be removed using the AgZnO/POMs nanocomposites through adsorption and photocatalysis. The kinetic analysis showed that both the adsorption and photocatalysis process conform to pseudo-second-order kinetics. In addition, the removal rate of AgZnO/POMs nanocomposites was found to be almost unchanged after 5 cycles of use. The bifunctional photocatalytic-adsorbent AgZnO/POMs nanocomposites with high stability and cycling performance have broad application prospects in the treatment of refractory organic dye wastewater containing triphenylmethane.

**Supplementary Information:**

The online version contains supplementary material available at 10.1186/s11671-021-03620-0.

## Introduction

With the development of industry, a large amount of toxic and harmful organic wastewater has caused a series of environmental problems, which seriously threaten human health [1–4]. Basic magenta (BM) is a kind of refractory organic pollutant containing triphenylmethane. BM is widely used as a colorant in industries such as textile and leather and also as a colorant for the stain of collagen, tuberculosis and muscle [5, 6]. It is urgently needed to be removed from the aqueous solution for the reason that BM poses a great threat to water resources due to its poor biodegradability, toxicity and carcinogenicity. According to the literature, the removal method of BM in aqueous solution is mainly adsorption [7, 8]. However, the application of BM dye adsorbents subjects to the disadvantages of low adsorption capacity, slow kinetic speed and low recovery potential. It is still a challenge to explore a cleaner and more effective method to remove BM from aqueous solution.

Polyoxometalates (POMs) are a class of promising adsorbents and have been applied in environmental protection because of their rich compositions and structures, high thermal stability, adjustable acidity and reversible redox properties [9–13]. As adsorbent, POMs have been used to synthesize a variety of materials to remove different dyes from aqueous solutions [14–17]. Liu's research group has reported Fe_3_O_4_/POMs nanomaterial with good adsorption performance for removal of cationic dyes, and Fe_3_O_4_/Ag/POMs nanomaterial with rapid removal of methylthionine chloride, indicating that more effective dye removal enhancement performance could be obtained by combining POMs and nanoparticles into a single entity through nanoengineering [18, 19].

**Scheme 1.**
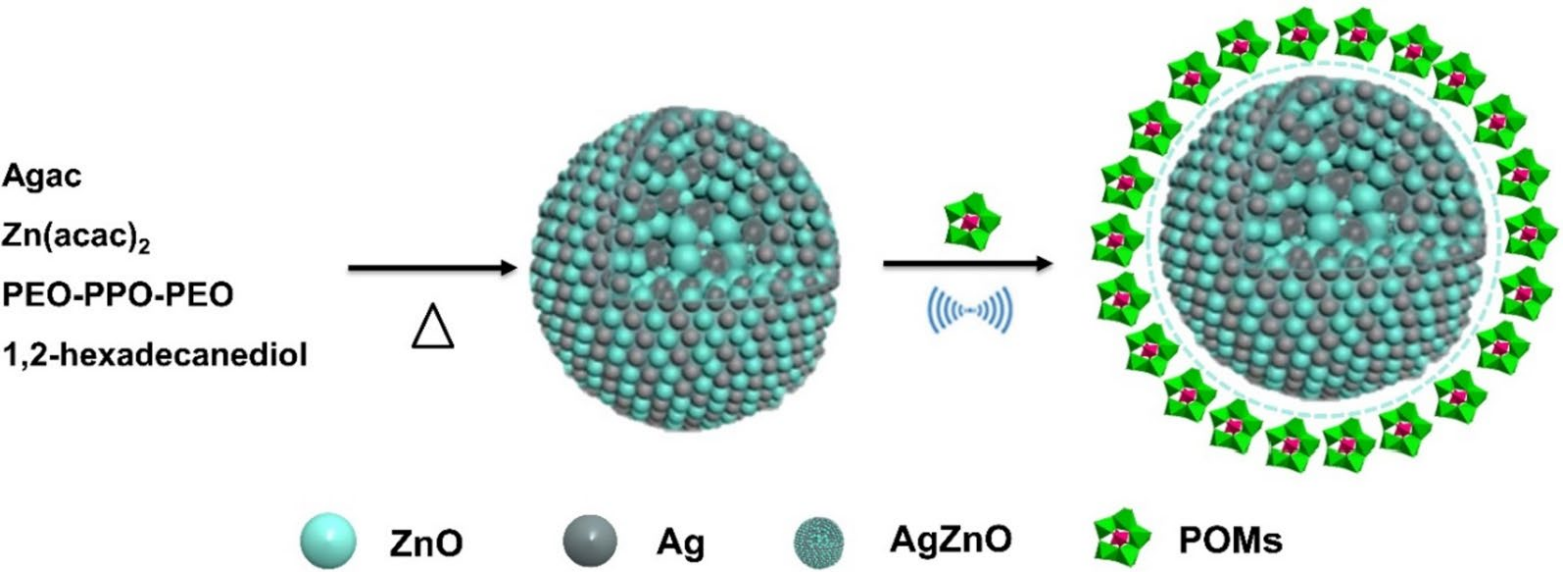
Diagram of synthesis of the AgZnO/POMs nanocomposites

AgZnO hybrid nanoparticles have excellent photocatalytic activity and are widely used in the field of photocatalysis. The addition of Ag improves photocatalytic capacity of AgZnO and the charge utilization efficiency and photochemical stability of ZnO [20–24]. Photocatalytic activity of AgZnO nanoparticles has photocatalytic effect on dyes in aqueous solution [25, 26]. In order to explore an effective and environmentally friendly method for removing BM dye in aqueous solution, in this paper, we combined AgZnO hybrid nanoparticles and POMs to obtain bifunctional photocatalytic-adsorbent AgZnO/POMs nanocomposites (Scheme 1). The removal experiments of BM demonstrated that photocatalytic-adsorbent AgZnO/POMs nanocomposites possessed both adsorption and photocatalytic effects on BM in aqueous solution with emerging high removal efficiency. The good adsorption, photocatalytic activity and reusability of the nanocomposites indicated that the bifunctional photocatalytic-adsorbent AgZnO/POMs nanocomposites are beneficial to protect the environment.

## Methods

The current study was aimed to improve the efficiency removal of BM by AgZnO/POMs nanocomposites.

### Materials

Silver acetate (Agac, 99%, J&K Scientific), Zinc(II) acetylacetonate (Zn(acac)_2_, 99.9%, J&K Scientific), PEO-PPO-PEO, n-octyl ether (99%), 1,2-hexadecanediol (90%), copper perchlorate (Cu(ClO_4_)_2_·6H_2_O, 98%), sodium molybdate dihydrate (Na_2_MoO_4_·2H_2_O, 99%), pyridinecarboxamide (C_6_H_6_N_2_O, 98%) and NaOH (98%) were purchased from Aladdin company (Shanghai, China). None of the materials were further purified.

### Instruments

The structure and morphology of the photocatalytic adsorbent AgZnO/POMs nanocomposites were analyzed by XRD (X’Pert Pro, Bruker, Germany) and TEM (JEM-2100 JEOL Ltd., Japan) including HRTEM. The optical properties of photocatalytic adsorbent AgZnO/POMs nanocomposites were characterized by UV–Vis (Hitachi U4100, Japan) and PL spectroscopy (Hitachi F7000, Japan). The FTIR spectra of nanocomposites were recorded using Avatar 360 FTIR spectrometer (Nicolet Company, USA). The XPS were performed on photoelectron spectrometer (Thermo Fisher Scientific ESCALAB 250XI, United States) Al K*α* X-ray used as the excitation source.

### Synthesis of Photocatalytic-Adsorbent AgZnO/POMs Nanocomposites

The AgZnO and polyoxometalates [Cu(L)_2_(H_2_O)_2_]H_2_[Cu(L)_2_P_2_Mo_5_O_23_]·4H_2_O (Cu-POMs) samples were synthesized using the method reported in the literature [19, 21]. Firstly, AgZnO hybrid nanoparticles were synthesized by nano-microemulsion method, 10 mL of octyl ether, Zn(acac)_2_ (0.0989 g), 1,2-hexadecanediol (0.6468 g), Agac (0.0259 g) and PEO-PPO-PEO (0.7874 g) were added to a three-necked flask, and the mixture was stirred. The mixture was heated to 125 °C, then the temperature was quickly raised to 280 °C, and the experiment was completed. When the temperature was cooled, the AgZnO hybrid nanoparticles were taken out and washed, obtaining pure AgZnO hybrid nanoparticles. Secondly, Cu-POMs was synthesized by hydrothermal method, and copper perchlorate (0.093 g), 2-pyridinecarboxamide (0.061 g) and 15 mL of deionized water were added to a beaker, stirred and mixed. When the temperature was cooled to room temperature, Na_2_MoO_4_·2H_2_O (0.24 g) and deionized water (10 mL) were added to the solution and mixed well, and pH was maintained at 3. The blue precipitate Cu-POMs was obtained by filtration. Thirdly, a mixture of reactants was obtained by adding 50 mg POMs powders and 5 mg AgZnO hybrid nanoparticles into beaker containing 5 mL water and 5 mL ethanol, ultrasonically treated to obtain a uniform liquid. This process combines the AgZnO hybrid nanoparticles with Cu-POMs to form nanostructures. Finally, the samples were dried to obtain a bifunctional AgZnO/POMs nanocomposite with both photocatalysis and adsorption effects.

### Dye Removal Experiment

The removal activity was researched by analyzing the removal efficiency of BM from aqueous solution. In the removal experimental study, a 36-W UV lamp (Philips, Netherlands, emitting mainly 365 nm) and a 500-W Xenon lamp were used as light source. The dye was dissolved in water to prepare 15 mg/L BM aqueous solution (room temperature condition, pH = 6.3). The 5 mg of nanocomposites was added to 40 mL (15 mg/L) BM solution for experiments. The solution was magnetically stirred at room temperature. At different time intervals, about 5 mL solution was removed and centrifuged for 3 min. The absorption peak intensity of BM at the maximum wavelength of 545 nm was analyzed by UV–Vis spectrophotometer.

### Statistical Analysis

Statistical analysis was compiled on the means of the results obtained from at least three independent experiments. All data were presented as means ± standard deviation and statistically compared using one-way analysis of variance (ANOVA). A *p* value less than 0.05 was considered statistically significant.

## Results and Discussion

### TEM Analysis of Photocatalytic Adsorbent AgZnO/POMs Nanocomposites

**Fig. 1:**
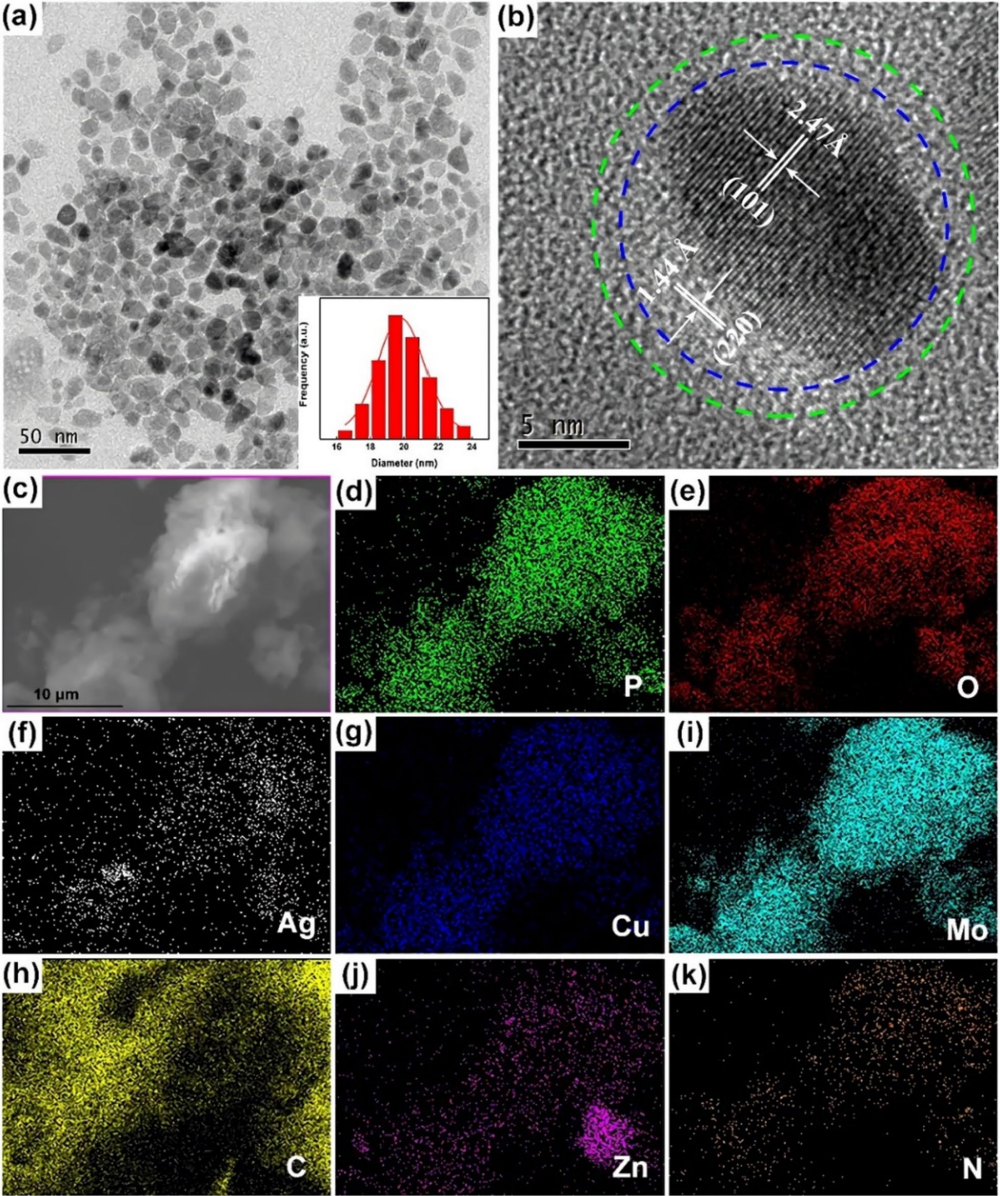
**a** TEM micrographs and illustration show particle size histogram of AgZnO/POMs nanocomposites, **b** HRTEM of single AgZnO/POMs, **c** STEM micrographs and **d**–**k** corresponding elemental mappings of AgZnO/POMs nanocomposites

The particle size distribution and morphology of photocatalytic-adsorbent AgZnO/POMs nanocomposites were analyzed by TEM and SEM. In Fig. 1a, the AgZnO/POMs nanocomposites are uniform particles size without agglomeration. By measuring the TEM micrographs of AgZnO/POMs nanocomposites, the histogram of particle size distribution was obtained. The average particle size of AgZnO/POMs nanocomposites was about 19.5 nm, which was consistent with the Gaussian distribution. Figure 1b shows the high resolution transmission electron microscopy (HRTEM) image of AgZnO/POMs. Apparently, the nanocomposites are distributed with highly regular lattices, in which the spacing of 1.44 Å corresponds to the Ag (220) plane, while the spacing of 2.47 Å is assigned to ZnO (101) plane. A spacing of about 1 nm between the blue dotted line and the green dotted line may be distributed with POMs [27]. Element mapping (Fig. 1c–k) confirmed the distribution of P, O, Ag, Cu, Mo, N, C and Zn in the AgZnO/POMs nanocomposites and showed that AgZnO and POMs existed simultaneously in AgZnO/POMs nanocomposites. The results confirmed the formation of photocatalytic adsorbent AgZnO/POMs nanocomposites.

### XRD Analysis of Photocatalytic Adsorbent AgZnO/POMs Nanocomposites

**Fig. 2:**
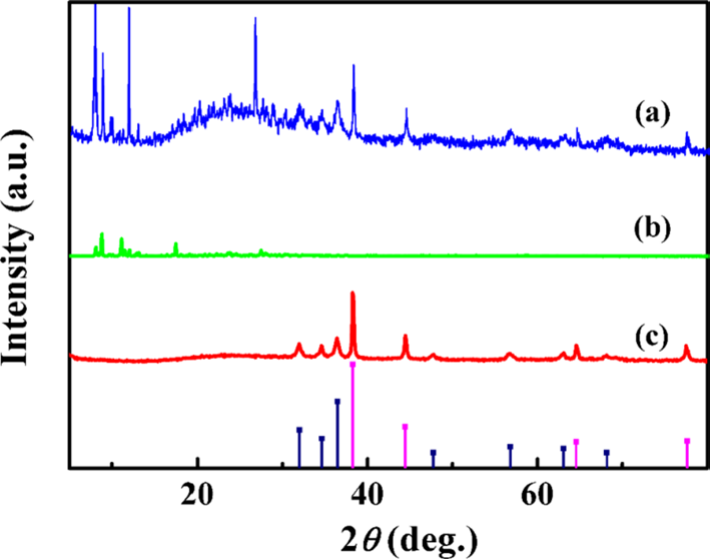
XRD patterns of **a** AgZnO/POMs nanocomposites, **b** POMs, **c** AgZnO hybrid nanoparticles (the purple and blue column charts are the column diagrams of Ag and ZnO labeled cards, respectively)

The structure of prepared photocatalytic adsorbent AgZnO/POMs nanocomposites was analyzed by XRD. In Fig. 2c, the diffraction peaks marked by the purple column diagrams of AgZnO hybrid nanoparticles at 38.2°, 44.4°, 64.6° and 77.4° correspond to the characteristic peaks of Ag (JCPDS No. 04-0783). The peaks marked by the blue column diagrams at 31.7°, 34.5°, 36.5°, 47.6°, 56.7°, 62.8° and 67.7° correspond to ZnO (JCPDS No. 36-1451) characteristic diffraction peaks. The peaks at 8.7°–30.7° in Fig. 2b are the diffraction peaks of POMs [19]. In the diffraction pattern of photocatalytic adsorbent AgZnO/POMs nanocomposites (Fig. 2a), the diffraction peaks of POMs (Fig. 2b) and AgZnO hybrid nanoparticles (Fig. 2c) reappear simultaneously. The results confirmed the formation of AgZnO/POMs nanocomposites.

### FTIR Analysis of Photocatalytic Adsorbent AgZnO/POMs Nanocomposites

**Fig. 3:**
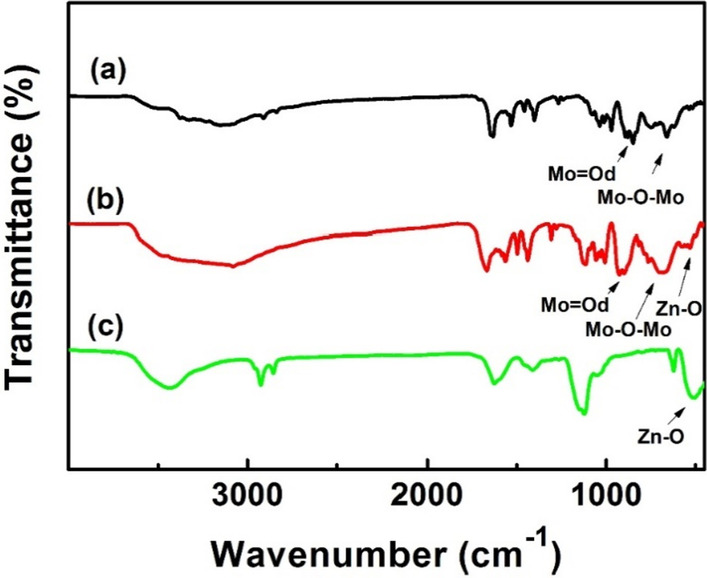
FTIR spectra of **a** POMs, **b** AgZnO/POMs nanocomposites and **c** AgZnO hybrid nanoparticles

The FTIR spectra of AgZnO/POMs nanocomposites, POMs, and AgZnO hybrid nanoparticles were depicted in Fig. 3a–c. As shown in Fig. 3a, the vibration peak at 3370 cm^−1^ is caused by the H_2_O hydrogen bond. The vibration peak appearing in the interval of 1680–1133 cm^−1^ is attributed to the ligand 2-pyridinecarboxamide. The stretching vibration of the P-O bond appears in the range of 1120–1008 cm^−1^ [28, 29]. The vibrational peaks at 905 cm^−1^ and 662 cm^−1^ are attributed to the *ν* (Mo–O_bridging_) bond and the *ν* (Mo–O_terminal_) bond, respectively [29]. The characteristic absorption peaks in POMs appear in the map of photocatalytic-adsorbent AgZnO/POMs nanocomposites. In Fig. 3c, the strong absorption at 512 cm^−1^ clearly reflects the vibration of the Zn–O bond, and the corresponding peak also appears in Fig. 3b [30]. The above characteristic absorption peaks also exist in the FTIR spectra of photocatalytic-adsorbent AgZnO/POMs nanocomposites (Fig. 3b), confirming that the nanocomposites were synthesized.

### XPS Analysis of Photocatalytic Adsorbent AgZnO/POMs Nanocomposites

**Fig. 4:**
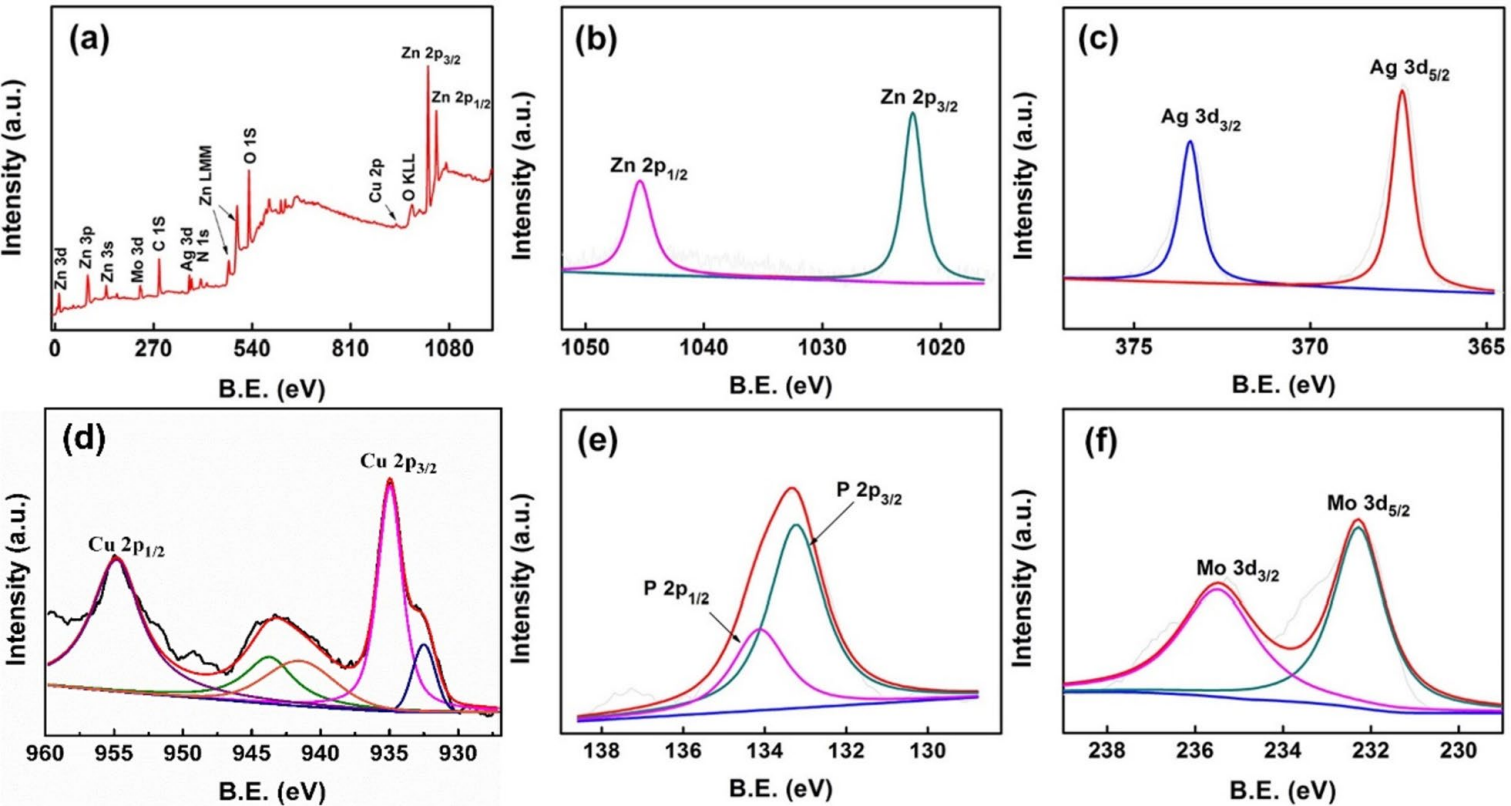
XPS spectra of AgZnO/POMs nanocomposites **a** full spectrum, **b** Zn 2*p* map, **c** Ag 3*d* map, **d** Cu 2*p* map, **e** P 2*p* map, **f** Mo 3*d* map

In Fig. 4, the XPS spectrum was calibrated using C1s (284.8 eV). The peaks of C, O, N, P, Zn, Mo, Cu and Ag can be observed from the full spectrum of XPS (Fig. 4a). In Fig. 4b, the AgZnO/POMs nanocomposites show two peaks of binding energy at approximately 1022 eV and 1045 eV, corresponding to the main regions of Zn 2*p*_3/2_ and Zn 2*p*_1/2_ [31]. The first peak is attributed to the Zn^2+^ ion in the anoxic zinc oxide [32]. The peaks at 367.2 eV and 373.2 eV (Fig. 4c) correspond to Ag 3d_5/2_ and 3d_3/2_ states of metal Ag. Compared with bulk silver (about 368.2 eV and 374.2 eV, respectively), the peaks of the Ag 3d state is significantly transferred to the lower value of AgZnO hybrid nanoparticles, which is attributed to contact between Ag and ZnO [33]. Figure 4d shows peaks at 934.9 eV and 954.7 eV, which are in the energy region of Cu 2p_3/2_ and Cu 2p_1/2_ attributed to Cu^2+^, indicating that Cu is mainly present in the form of Cu^2+^ [34, 35]. Figure 4e shows peaks at 133.2 and 134.1 eV, corresponding to the P–O peaks of P 2*p*_3/2_ and P 2*p*_1/2_, respectively [36]. In Fig. 4f, shows peaks at 235.8 and 232.3 eV, corresponding to the main regions of Mo 3*d*_3/2_ and Mo 3*d*_5/2_, respectively, indicating that the valence of Mo is mainly Mo^6+^ [37]. The analysis shows that AgZnO/POMs nanocomposites contain AgZnO and POMs.

### UV–Vis Analysis of Photocatalytic Adsorbent AgZnO/POMs Nanocomposites

**Fig. 5:**
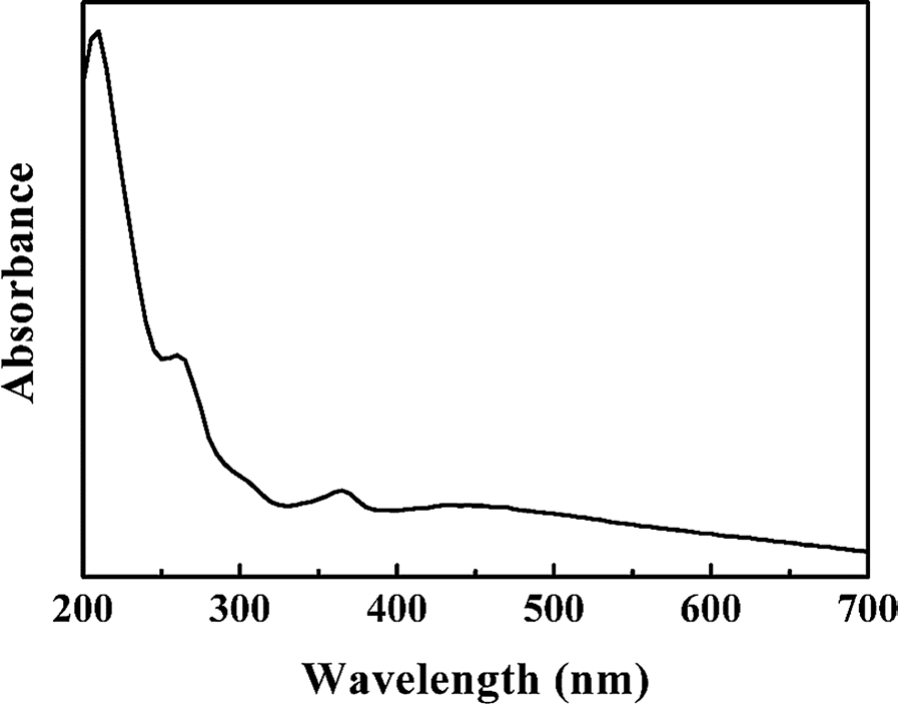
UV–Vis absorption spectrum of photocatalytic-adsorbent AgZnO/POMs nanocomposites

UV–Vis absorption spectrum of photocatalytic-adsorbent AgZnO/POMs nanocomposites in aqueous solution is shown in Fig. 5. The AgZnO/POMs nanocomposites have four absorption bands at 209 nm, 260 nm, 365 nm and 380–420 nm, respectively. The absorption band at 365 nm is the characteristic absorption band of ZnO [21]. The absorption at 380–420 nm reveals the hybridization of ZnO with Ag and the interfacial electron interaction between Ag and ZnO [38]. The absorption bands at 209 nm and 260 nm are attributed to POMs because of electron transfer of O_terminal_ → Mo and O_bridging_ → Mo in POMs [19]. The results show that the AgZnO/POMs nanocomposites have excellent optical properties.

### PL Analysis of Photocatalytic Adsorbent AgZnO/POMs Nanocomposites

**Fig. 6:**
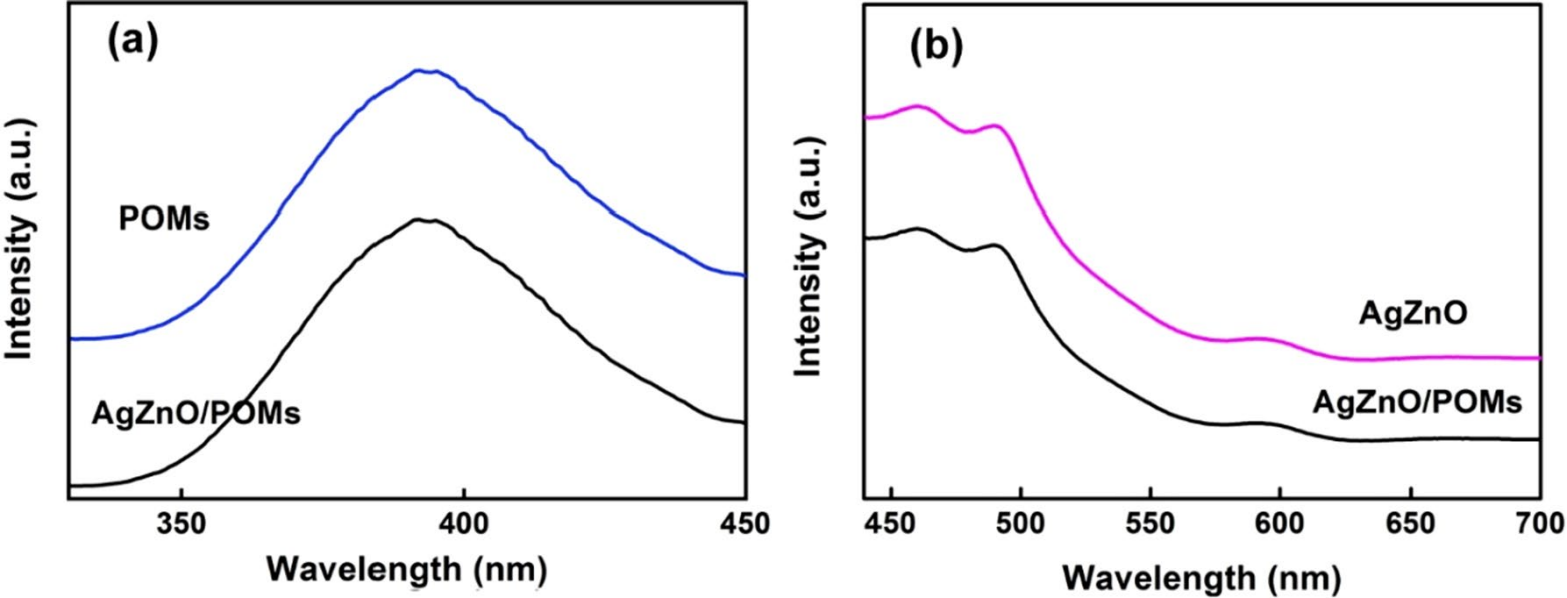
**a** Solid PL emission spectra of POMs and AgZnO/POMs with excitation wavelength *λ*_ex_ = 241 nm, **b** Solid PL emission spectra of AgZnO and AgZnO/POMs with excitation wavelength *λ*_ex_ = 380 nm

The solid fluorescence emission spectra of photocatalytic-adsorbent AgZnO/POMs nanocomposites were detected under the excitation wavelength of 241 nm (Fig. 6a) and 380 nm (Fig. 6b), respectively. As shown in Fig. 6a, AgZnO/POMs nanocomposites have an emission peak at 393 nm, corresponding to the solid-state fluorescence emission peaks at 393 nm of POMs [39]. Figure 6b AgZnO/POMs nanocomposites shows three emission peaks at 465 nm, 489 nm and 596 nm corresponding to the emission peaks of AgZnO hybrid nanoparticles, respectively. The blue light emission peaks at 465 nm and 489 nm are usually caused by photo-generated holes of ZnO and the oxygen vacancies occupied by the nanocomposites [40]. The emission at about 596 nm is generally thought to be caused by the recombination of electrons and valence band holes in the deep defect layer of ZnO [41]. The results show that the AgZnO/POMs nanocomposites have excellent optical properties.

## Removal of BM

**Fig. 7:**
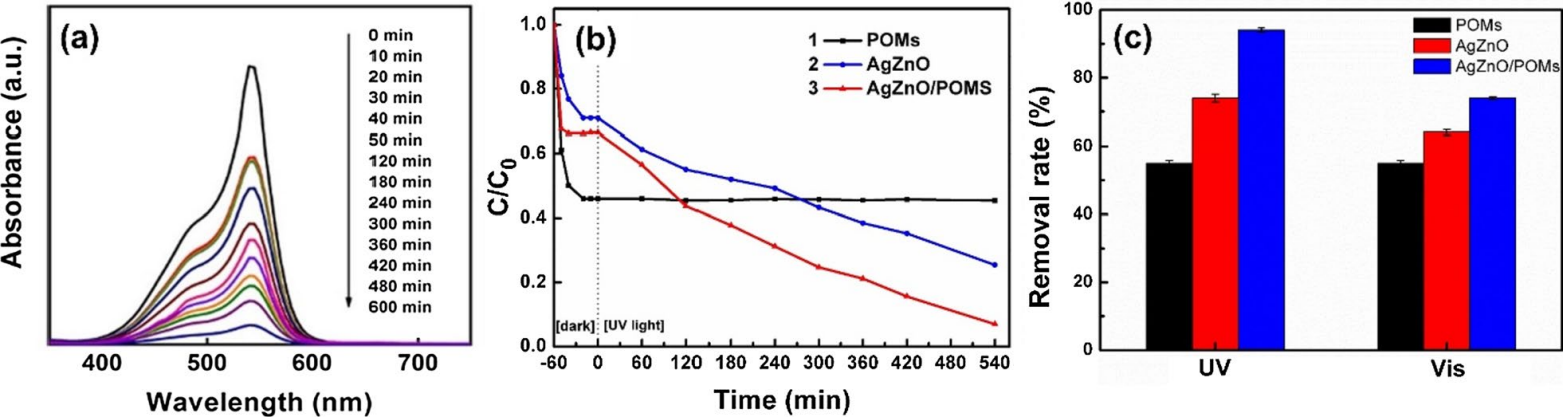
**a** UV–Vis absorption spectra of BM solution containing the AgZnO/POMs nanocomposites, **b** removal curves of different materials for removing BM, curve: (1) POMs, (2) AgZnO, (3) AgZnO/POMs nanocomposites (The experiment was repeated three times), **c** Histogram of the removal of BM by POMs, AgZnO and AgZnO/POMs nanocomposites under UV and Vis irradiation (The experiment was repeated three times)

The adsorption and photocatalytic activities of AgZnO/POMs nanocomposites were studied by removing BM from aqueous solution. In the BM removal experiment, the dosage of AgZnO/POMs and concentration of BM are very significant parameter. Through a series of optimization experiments, the most suitable of AgZnO/POMs dosage and BM concentration are 5 mg and 15 mg/L, respectively (Additional file 1: Fig. S1). Figure 7a is the UV–Vis absorption spectra of BM solution containing the AgZnO/POMs nanocomposites at different intervals. Figure 7b shows a comparative study for removing BM in the presence of (1) POMs, (2) AgZnO and (3) AgZnO/POMs nanocomposites, in which, the ordinate is C/C_0_, where C is the corresponding concentration of BM at different time intervals and C_0_ is the original concentration of BM. It can be observed in combination with Fig. 7a and b that the absorption peak strength of BM gradually decreases in 0–30 min, remains unchanged in 30–50 min for reaching adsorption equilibrium under stirring in the dark, and then after 50 min decreases with the increase in UV-light irradiation, indicating the adsorption and photocatalysis activities of AgZnO/POMs nanocomposites. For verifying the photocatalytic-adsorption synergistic effect, the removal experiment of BM from aqueous solution was investigated using AgZnO/POMs, POMs and AgZnO with amount of 5 mg. The removal rate was 94.13% ± 0.61, 55.27% ± 0.83 and 73.77% ± 1.17, respectively. The removal rate of BM decreased significantly using only POMs adsorbent or only AgZnO photocatalyst compared with photocatalytic-adsorbent AgZnO/POMs (Fig. 7b). This is mainly due to the synergistic effect of AgZnO and POMs, and the synergistic effect can be divided into two aspects: (1) In AgZnO/POMs core–shell structure, the shell layer (POMs) can adsorb BM molecules extremely easily. Adsorbed BM molecules are confined around the core (AgZnO), which is beneficial for the next photocatalytic degradation; (2) the oxygen-rich structures of POMs can prevent the recombination of photogenerated *e*^*−*^ and *h*^+^ and further improve the separation efficiency. Figure 7c shows a comparative histogram of the removal of BM by POMs, AgZnO and AgZnO/POMs nanocomposites under UV-light and Vis irradiation, respectively. No matter under UV or visible light irradiation, the photocatalytic-adsorbent AgZnO/POMs have higher removal efficiency than the adsorbent POMs and photocatalyst AgZnO. The removal rate of AgZnO/POMs for removing BM is 94.13% ± 0.61, which is much higher than that of POMs (55.27% ± 0.83) and AgZnO (73.77% ± 1.17) under UV-light irradiation. Compared to the recently reported works about removal of BM, the AgZnO/POMs demonstrate a better performance than the other cases (Additional file 1: Table S1). In addition, except for BM, AgZnO/POMs can also effectively remove gentian violet (removal rate: 90.30% ± 0.58) and methylene blue (removal rate: 89.00% ± 1.00) from aqueous solution (Additional file 1: Fig. S2).

**Fig. 8:**
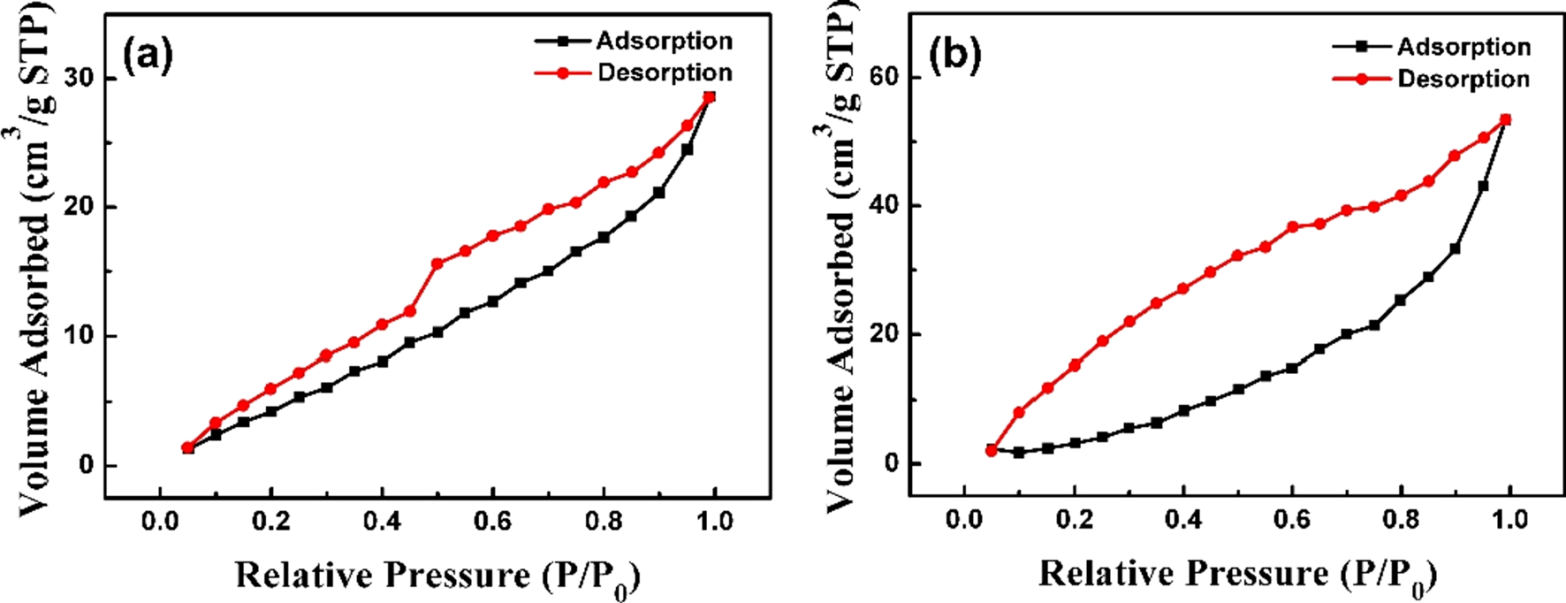
**a** N_2_ adsorption–desorption isotherm of AgZnO hybrid nanoparticles, **b** N_2_ adsorption–desorption isotherm of AgZnO/POMs nanocomposite

The N_2_ adsorption–desorption isotherms of AgZnO nanoparticles and photocatalytic-adsorbent AgZnO/POMs nanocomposites were determined using the automatic physical/chemical adsorption apparatus. In Fig. 8, both samples showed typical type IV isotherms, indicating the presence of mesoporous structures [42]. According to the analysis results of relative position and height of hysteresis loops (Fig. 8), the specific surface area (BET) of AgZnO nanoparticles (Fig. 8a) is 28.682 m^2^/g and the BET of AgZnO/POMs nanocomposites (Fig. 8b) is 33.535 m^2^/g. The results indicate that the AgZnO/POMs nanocomposites obtained by the combination of the two have a higher specific surface area, which correspond to the enhanced adsorption performance of the composite under dark conditions.

The pseudo-first-order and pseudo-second-order kinetic models were used to fit the experimental data of AgZnO/POMs nanocomposites.

1 $${\text{ln}}\left( {q_{e} - q_{t} } \right) = {\text{ln}}q_{e} - k_{1} t$$

2 $$\frac{t}{{q_{t} }} = \frac{1}{{k_{2} \left( {q_{e} } \right)^{2} }} + \frac{t}{{q_{e} }}$$

In (1) and (2), *q*_0_ is adsorption amount at *t* = 0, *q*_e_ is equilibrium adsorption amount, *q*_t_ is adsorption amount at time *t*, *k*_1_ and *k*_2_ are the pseudo-first-order and pseudo-second-order kinetic rate constants, respectively.

**Fig. 9:**
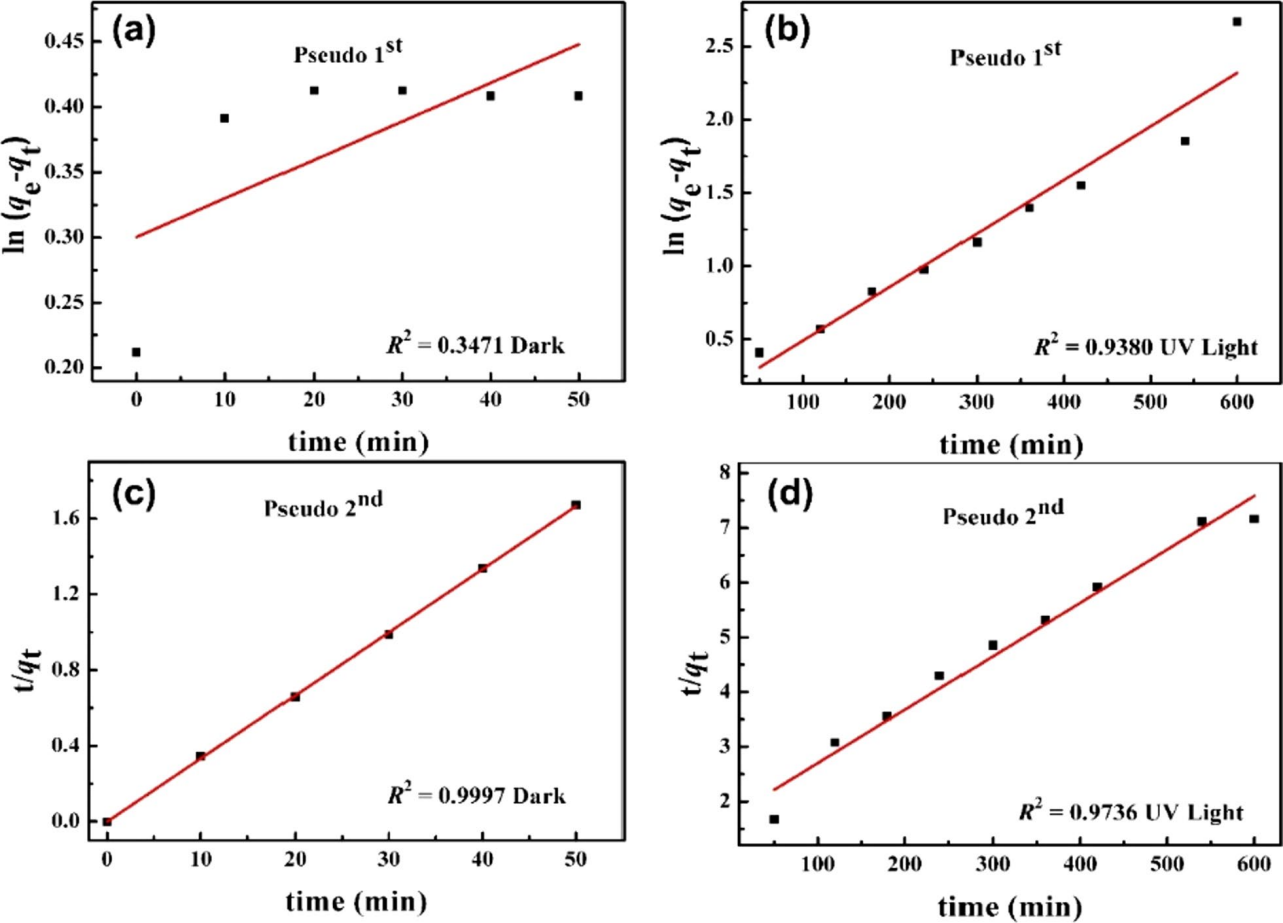
The kinetic plots for removing BM by AgZnO/POMs nanocomposites, **a** and **b** pseudo-first-order kinetics, **c** and **d** pseudo-second-order kinetics

The kinetic plots of removing BM by AgZnO/POMs nanocomposites are shown in Fig. 9, and the results are shown in Table 1. The correlation coefficient (*R*^2^) of pseudo-second-order model (0.9997 and 0.9736) was higher than that of pseudo-first-order model (0.3471 and 0.9380) under dark and UV light, respectively. Furthermore, another parameter called the residual sum of squares (SSR) which shows the error value is smaller in the pseudo-second-order kinetic model. Therefore, it can be indicated that both the adsorption process and the photocatalysis process of removing BM by AgZnO/POMs nanocomposites followed the pseudo-second-order kinetics. The results demonstrate that the removal rate of AgZnO/POMs nanocomposites is mainly due to the chemical adsorption and electron transfer ability of the composites [27, 43].

**Table 1:** Kinetic correlation coefficients (*R*^2^) fitting parameters

	Pseudo-first-order		Pseudo-second-order	
	*R*^2^	SSR	*R*^2^	SSR
Dark	0.3471	0.62	0.9997	0.0004
UV light	0.9380	0.21	0.9736	0.20

**Fig. 10:**
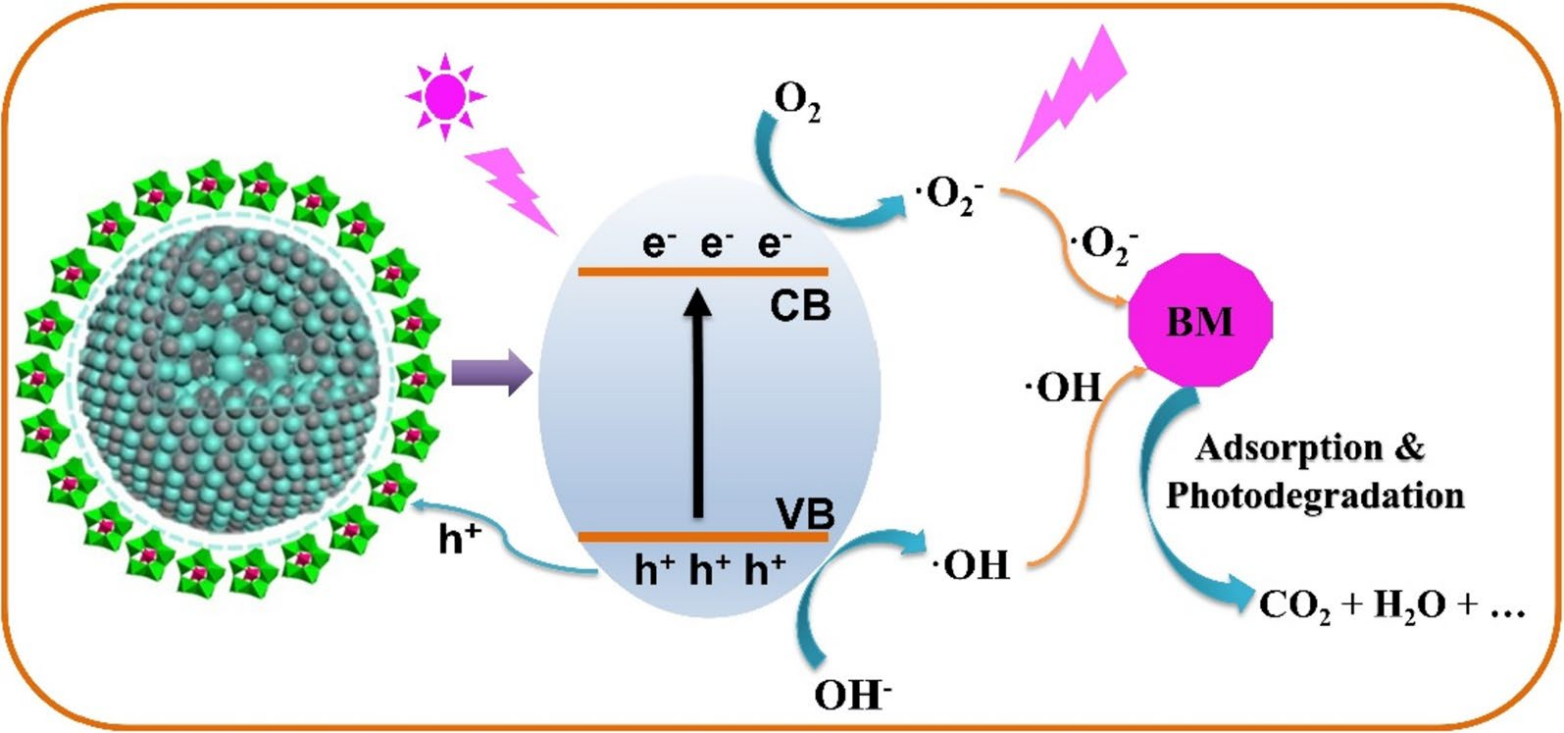
Schematic illustration of removal of BM by AgZnO/POMs nanocomposites

The removal of BM can be attributed to two factors: first, POMs as adsorbent to adsorb BM from aqueous solution; second, adsorbed BM molecules can be degraded via AgZnO photocatalyst. As shown in Fig. 10, when BM molecules are adsorbed and confined around the AgZnO via POMs, AgZnO nanoparticles are excited by UV light, the photogenerated *e*^*−*^ and hole (*h*^+^) will be produced by ZnO (Ag acts as an electron acceptor). In addition, the oxygen-rich structures of POMs are also beneficial for preventing the recombination of photogenerated *e*^*−*^ and *h*^+^ and thus further improve the separation efficiency. The photogenerated *e*^*−*^ can react with chemisorbed oxygen molecular to form superoxide radicals (˙O_2_^−^). At the same time, the *h*^+^ in the valence band of ZnO reacts with hydroxyl groups to form hydroxyl radicals (˙OH). The *h*^+^, ˙OH and ˙O_2_^−^ produced in the process of photocatalysis are crucial substances for degradation of BM [19, 27, 44]. These created intermediates possess highly reactive (namely strong oxidation) and have the ability to oxidize the BM dye into CO_2_, H_2_O and some corresponding simple compounds. As a result, the removal rate of AgZnO/POMs nanocomposites is greatly improved by the combination of AgZnO and POMs into a whole nanoengineering. The photocatalytic-adsorbent AgZnO/POMs nanocomposites are expected to be a new type of dye remover, which can remove efficiently aromatic organic dyes from water pollution, especially for BM. In addition, to further prove the generation of free radical, reactive oxygen species (ROS) scavenger was utilized to eliminate ROS during the photocatalytic process. 1, 4-Benzoquinone (BQ) and isopropanol (IPA) are free radical scavenger. The BQ and IPA can rapidly scavenge O_2_^−^ radical and ˙OH radical, respectively [45, 46]. When free radical scavenger (BQ and IPA) was added into a removal experiment of BM, removal rate of BM significantly decreases. For BQ + AgZnO/POMs, removal rate of BM from 94.13% ± 0.61 drops to 52.17% ± 0.76. For IPA + AgZnO/POMs, removal rate of BM from 94.13% ± 0.61 drops to 57.70% ± 0.70. Such results imply the key active substances (˙OH and ˙O_2_^−^) can be generated in the process of removing BM from AgZnO/POMs nanocomposites (Additional file 1: Fig. S3).

**Fig. 11:**
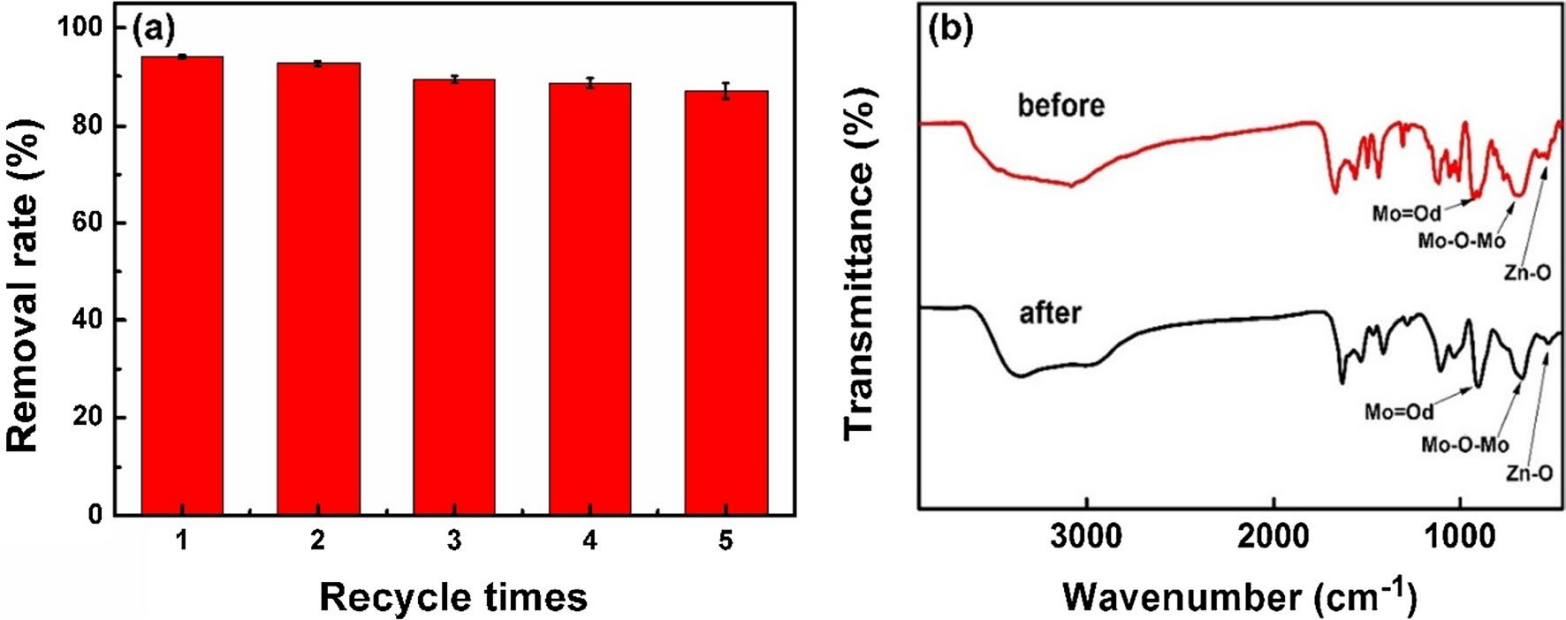
**a** Histogram of recycle removal BM for 5 cycles (each cycle experiment was repeated three times), **b** Comparison of FTIR spectra of AgZnO/POMs nanocomposites before and after 5 cycles

To investigate the reproducibility of the nanocomposites for removing BM, we collected and washed the AgZnO/POMs nanocomposites. The collected nanocomposites were used to remove BM via five repeated experiments under the same reaction conditions. As shown in Fig. 11a, the removal rate of BM in AgZnO/POMs nanocomposites decreased by only 7.33% (from 94.13% ± 0.61 to 86.80% ± 1.58) after five cycles, the slight reduction might correspond to the loss of AgZnO/POMs nanocomposites during washing (average recovery rate of AgZnO/POMs is 96.3%). Figure 11b shows that the FTIR spectrum of the AgZnO/POMs nanocomposites before and after BM removal is similar. It could be proved that the nanocomposites have good stability and light corrosion of resistance (Scheme 1).

## Conclusions

In conclusion, the photocatalytic-adsorbent AgZnO/POMs nanocomposites were synthesized by combining AgZnO hybrid nanoparticles and POMs. The TEM and HRTEM showed that AgZnO/POMs nanocomposites were uniform with narrow particle size distribution and without agglomeration. The bifunctional photocatalytic-adsorbent AgZnO/POMs nanocomposites could effectively remove refractory BM from aqueous solution with removal efficiency of 94.13% ± 0.61 by adsorption and photocatalysis. The adsorption process and the photocatalytic process of AgZnO/POMs nanocomposites for removing BM followed the pseudo-second-order kinetics. The removal efficiency of AgZnO/POMs nanocomposites was found to be almost unchanged after 5 cycles of use, demonstrating that the nanocomposites have well stability in BM in aqueous solution. The FTIR spectra of AgZnO/POMs nanocomposites before and after BM removal are almost no change, further indicating the stability of nanocomposites. The bifunctional photocatalytic-adsorbent AgZnO/POMs nanocomposites have potential applications in the treatment of refractory organic dye wastewater containing triphenylmethane.

## Supplementary Information

**Additional file 1.** Synergistic photocatalytic-adsorption removal of basic magenta effect of AgZnO/polyoxometalates nanocomposites.

## Data Availability

Data sharing is not applicable to this article as no datasets were generated or analyzed during the current study.

## Abbreviations

AgZnO/POM: AgZnO/polyoxometalates
POMs: Polyoxometalates
HL: C_6_H_6_N_2_O
M: Basic magenta
Agac: Silver acetate
Zn(acac)_2_: Zinc(II) acetylacetonate
PEO-PPO-PEO: Triblock copolymer poly(ethylene glycol)-block-poly(propylene glycol)-block-poly(ethylene glycol)
Cu-POMs: [Cu(L)_2_(H_2_O)_2_]H_2_[Cu(L)_2_P_2_Mo_5_O_23_]·4H_2_O
TEM: Transmission electron microscopy
HRTEM: High-resolution transmission electron microscopy
SEM: Scanning electron microscope
XRD: X-ray powder diffraction
FTIR: Fourier transform infrared
XPS: X-ray photoelectron spectra
UV–vis: Ultraviolet–visible spectra
PL: Photoluminescence spectra
BET: Specific surface area
*R*^2^: Correlation coefficient
SSR: Residual sum of squares
BQ: 1, 4-Benzoquinone
IPA: Isopropanol
