# Supplementary material for: Synergistic Photocatalytic-Adsorption Removal of Basic Magenta Effect of AgZnO/Polyoxometalates Nanocomposites
Source: Nanoscale Res Lett. 2021 Nov 10;16:163. doi: 10.1186/s11671-021-03620-0 (PMC8581081; doi:10.1186/s11671-021-03620-0)
Supplement: Supplementary file 1 — Additional file 1. Synergistic photocatalytic-adsorption removal of basic magenta effect of AgZnO/polyoxometalates nanocomposites. [file 11671_2021_3620_MOESM1_ESM.docx]

**Additional file 1**

**Synergistic photocatalytic-adsorption** **removal of** **basic magenta effect of AgZnO/polyoxometalates nanocomposite**

Heyun Tian, Jie Luo, Ke Zhang, Chenguang Ma, Yiyi Qi, Shixia Zhan, Xiao Liu*, Hongling Liu*, and Mingxue Li*

*Key Lab of Polyoxometalate Chemistry of Henan Province, Institute of Molecular and Crystal Engineering, School of Chemistry and Chemical Engineering,* *Henan University, Kaifeng 475001, China.*

*E-mail:* [*hlliu@henu.edu.c*n](mailto:hlliu@henu.edu.cn)


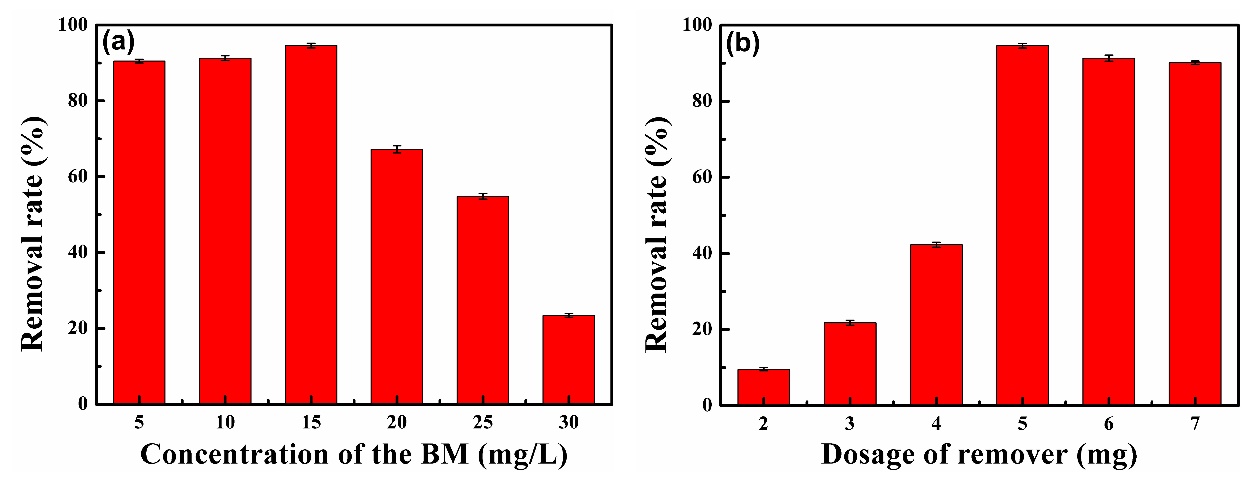


**Figure S1**. (a) Effect of BM dye concentration on the removal rate (The experiment was repeated three times), (b) Effect of catalyst dosage on the BM removal rate (The experiment was repeated three times).





**Figure S2**. Histogram of removal rate of basic magenta, gentian violet and methylene blue by AgZnO/POMs nanocomposites (The experiment was repeated three times).





**Figure S3.** Removal curves of BQ+AgZnO/POMs, IPA+AgZnO/POMs, and AgZnO/POMs for removing BM (The experiment was repeated three times).

**Table S1.** Comparison of removal rate of different removers for basic magenta.

| Remover | Removal rate | Method | Reference |
| --- | --- | --- | --- |
| graphene oxide/zinc oxide | 92 % | photocatalysis | [1] |
| pacara earpod tree  seeds | 66 % | adsorption | [2] |
| calcined mussel shell material | 91 % | adsorption | [3] |
| double functional groups modified bagasse | 91 % | adsorption | [4] |
| RGO-modified Natural luffa sponge | 86 % | adsorption | [5] |
| AgZnO/POMs | 94 % | adsorption and photocatalysis | this work |

**References**

1. Z. Durmus, B. Z. Kurt, A. Durmus, Synthesis and characterization of graphene oxide/zinc oxide (GO/ZnO) nanocomposite and its utilization for photocatalytic degradation of basic fuchsin dye, ChemistrySelect, 4 (2019), 271-278. https://doi.org/10.1002/slct.201803635.
2. L. O. S. Yamil, G. Jordana, S. R. Glaydson, C. L. Éder, L. S. O. Marcos, S. P. F. Dison, S. N. Matias, A. Daniel, L. D. Guilherme, Utilization of Pacara Earpod tree (Enterolobium contortisilquum) and Ironwood (Caesalpinia leiostachya) seeds as low-cost biosorbents for removal of basic fuchsin, Environmental Science and Pollution Research, 27 (2020) 33307-33320. https://doi.org/10.1007/s11356-020-09471-z.
3. M. El Haddad, Removal of Basic Fuchsin dye from water using mussel shell biomass waste as an adsorbent: Equilibrium, kinetics, and thermodynamics, Journal of Taibah University for Science, 10 (2016) 664-674. https://doi.org/10.1016/j.jtusci.2015.08.007.
4. R. Zhou, J. Yu, R. Chi, Simultaneous removal of cationic and anionic dyes from aqueous solution by double functional groups modified bagasse, Water Science and Technology, 82 (2020) 2159-2167. https://doi.org/10.2166/wst.2020.486.
5. S. Li, M. Tao, Y. Xie, Reduced graphene oxide modified luffa sponge as a biocomposite adsorbent for effective removal of cationic dyes from aqueous solution, Desalination and Water Treatment, 57 (2016) 20049-20057. https://doi.org/10.1080/19443994.2015.1106344.
